# Optimizing Curriculum Integration: Development and Validation of the Tool of Integration Practices (TIP) in Health Professions Education - A Mixed Methods Study

**DOI:** 10.12669/pjms.42.9.14806

**Published:** 2026-09

**Authors:** Syeda Rabbab Hasan, Madiha Sajjad, Rehan Ahmed Khan

**Affiliations:** 1 Syeda Rabbab Hasan, FCPS, FOrth RCSEd, MOrth RCSEd, MHPE, BDS Associate Professor, Department of Orthodontics, Shifa College of Dentistry, Shifa Tameer-e-Millat University, Islamabad, Pakistan; 2 Madiha Sajjad, FCPS, MHPE, MBBS Professor, Department of Pathology, Islamic International Medical College, Riphah International University, Rawalpindi, Pakistan; 3 Rehan Ahmed Khan, PhD-HPE, MHPE, FCPS, FRCS, MBBS Dean Riphah Institute of Assessment, Professor, Department of Surgery, Islamic International Medical College, Riphah International University, Rawalpindi, Pakistan

**Keywords:** Construct validity, Curriculum integration, Integration practices, Level of Integration, Reliability, Tool development

## Abstract

**Objective:**

Curriculum integration is increasingly adopted in medical and dental education, yet only a few validated tools exist to measure it objectively. Existing curriculum integration frameworks, including Harden’s ladder, are primarily descriptive and challenging to apply for systematic evaluation. Therefore, this study aimed to develop a valid and reliable tool to assess the level of integration in medical and dental curricula.

**Methodology:**

A multiphase mixed-method approach was employed over nine months from July 2023 to March 2024 at Riphah International University, Pakistan. Literature review identified eight domains representing curriculum integration, each structured on a five-point scale informed by Harden’s integration ladder. Content validity was established through expert reviews (n = 17), followed by cognitive interviews with faculty (n = 6) to obtain response process validity. Medical and dental faculty (n = 150) participated in pilot testing. Internal structure validity evidence was evaluated by Exploratory factor analysis (EFA) and confirmatory factor analysis (CFA). Reliability was assessed using Cronbach’s alpha.

**Results:**

Content validation yielded a seven-domain instrument with a mean content validity index of 0.89 and an average content clarity score of 3.46. Cognitive interviews led to minor revisions in four items. The final tool demonstrated good reliability (Cronbach’s alpha = 0.88). EFA supported a one-factor model, confirmed by CFA (GFI = 0.96, RMSEA = 0.052, normed χ² = 1.4), indicating a good model fit.

**Conclusion:**

The Tool of Integration Practices (TIP) demonstrated adequate validity and reliability. It provides a practical, psychometrically sound framework for assessing and comparing curriculum integration practices based on faculty perceptions at the course and program levels in health professions education.

## INTRODUCTION

Integrated curriculum is recognized for its potential to enhance the preparedness of future healthcare professionals and is therefore a widely adopted strategy for health professions education.^1^-^4^ It is based on the principles of Cognitive Learning, Constructivism, and Adult Learning theories.^2^ Research evidence indicates that students in integrated curriculum programs demonstrate better performance in clinical examinations, make earlier and more confident career decisions, and exhibit greater readiness for professional practice compared to those in traditional programs.^5^

Despite decades of curricular reforms, the degree to which integration is genuinely achieved within programs often remains uncertain.^4^-^6^ The diversity of integration models, horizontal, vertical, spiral, and theme-based, combined with contextual differences among institutions, makes it difficult to interpret and compare curricula meaningfully.^2^,^7^ Many institutions describe their curricula as “integrated,” but in practice, the depth and coherence of integration vary considerably. It can be influenced by institutional governance, faculty collaboration, and the availability of resources. Evaluations for the degree of curricular integration are often based on subjective interpretation rather than empirical evidence.^8^-^11^ The absence of standardized, objective criteria to assess integration practices has created a persistent gap between what is claimed and what is actually implemented in curriculum design and delivery within an institution.^3^,^4^,^10^,^11^

Over the years, several frameworks have sought to define and classify integration within curricula. Among these, Harden’s Integration Ladder remains the most widely recognized. It consists of eleven progressive levels of integration from isolated subject teaching to transdisciplinary curricula.^12^ Harden’s model is itself inspired by earlier works of Jacobs, Fogarty, and Drake.^12^ While conceptually influential, these models remain largely descriptive and theoretical, lacking the operational precision required for objective evaluation.^3^,^4^

Harden’s ladder assumes a linear and sequential evolution of integration, yet in practice, integration is dynamic, multidimensional, and context-dependent.^3^,^13^ Different domains of a curriculum, such as learning objectives, planning, delivery, and assessment, may simultaneously reflect varying levels of integration. A program may, for instance, employ integrated teaching strategies while retaining discipline-based assessments, or achieve thematic coherence in preclinical years but limited integration during clinical training. Another limitation lies in the terminology and complexity of Harden’s framework. The eleven steps are defined through nuanced terms such as “sharing,” “correlation,” or “complementary,” that, while conceptually logical, are difficult for educators to remember, distinguish, or communicate in practice.^3^ Thus, highlighting the need for a simplified and measurable classification system that retains conceptual rigor but is easier to operationalize across diverse institutional settings.^4^

Efforts to address these limitations have led to the development of a few instruments to measure curricular integration.^4^ While these tools have contributed to the field, they largely rely on perceptions of integration captured through generic rating scales that may not adequately reflect the complexity of integration practices within a curriculum. Furthermore, some are limited to evaluating individual teaching sessions rather than the curriculum as a whole.^4^ As a result, there remains a need for a valid and reliable instrument that can capture the full depth and complexity of curriculum integration at the course or program level across its essential domains.^4^ To address this critical gap, we aimed to develop and validate the Tool of Integration Practices (TIP), an instrument intended to evaluate the level of integration within medical and dental curricula. We hypothesized that TIP would demonstrate acceptable reliability and provide validity evidence across content, response process, and internal structure domains.

## METHODOLOGY

A multiphase mixed-method research design was followed.^14^ This study was conducted at Riphah International University, Pakistan, from July 2023 to March 2024. The study followed Messick’s unified framework for validity.^14^ The phases of tool (TIP) development are shown in Fig.1.

**Fig.1:**
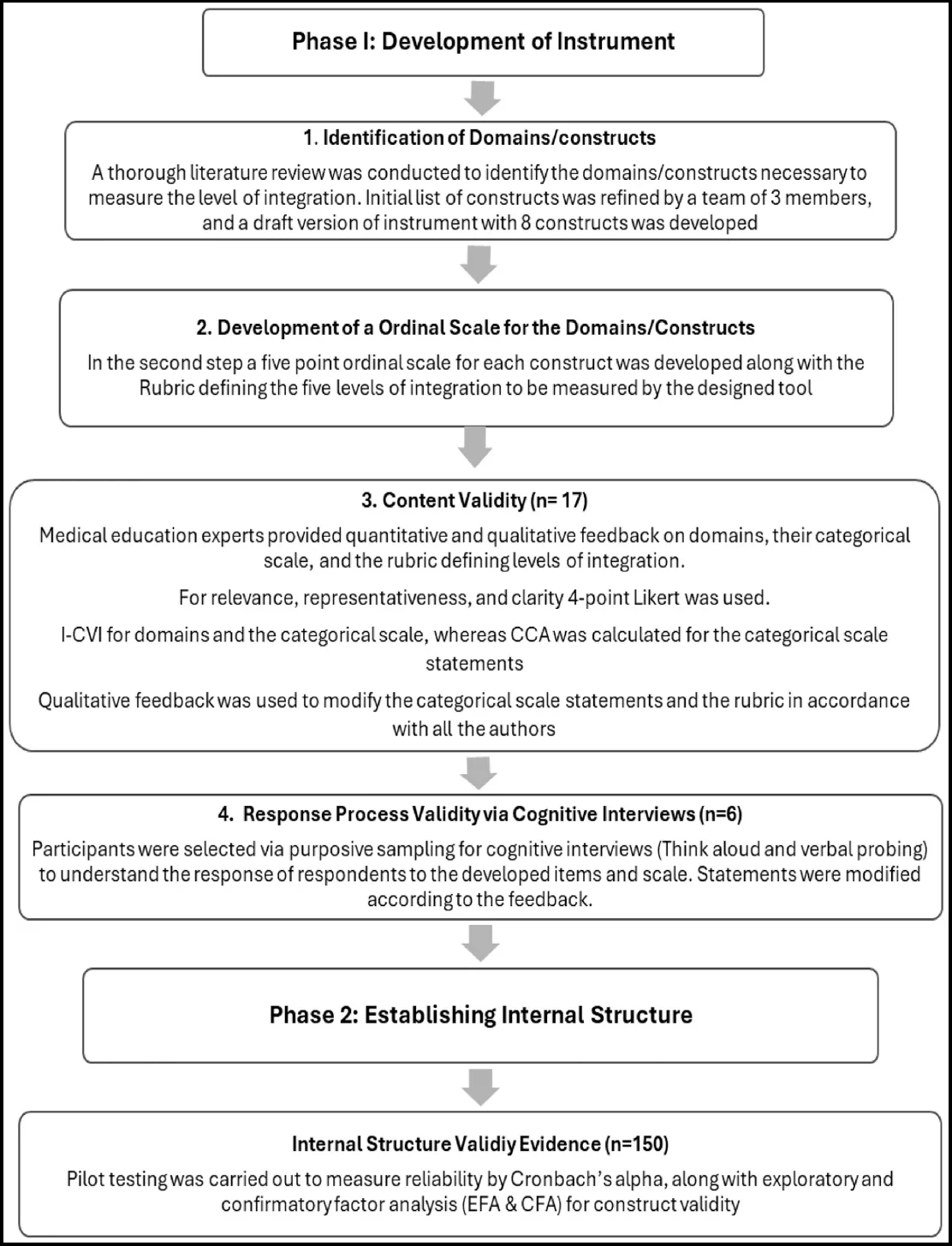
Phases for Development and Validation of the Tool of Integration Practices (TIP).

### Ethical approval:

It was obtained from the Ethical Review Committee of Islamic International University Appl. # Riphah/IRC/23/3030; dated January 27, 2023. At each stage of the research, written informed consent was taken from all the participants, and their confidentiality was ensured.

### Phase I- Development of the Instrument:

### Identification of Domains and Construction of an Ordinal Scale and a Rubric:

### Participants, materials, and procedures:

An extensive literature review was conducted to identify and define the key domains representing integration practices within a curriculum^2^ (Supplementary Table-IS). Based on these domains, an analytical rubric was developed to describe five progressive levels of curricular integration^2^,^12^,^15^ (Supplementary Table-IIS). For each domain, a five-point ordinal scale with descriptive anchors was constructed by systematically mapping relevant descriptors from Harden’s integration ladder to the corresponding levels of the rubric.

**Table-IS:** Domains identified to assess the level of integration.

Domains	Definition
Curricular aims and objectives	The intended outcomes and goals of a curriculum include explicit knowledge, skills, and attitudes that the learners are expected to obtain or develop through their educational journey.
Consultation in course planning prior to delivery	This involves participation and feedback from various stakeholders, including colleagues, experts, and learners, to enhance the quality and effectiveness of the course.
Representation of content in timetables	The organization and allocation of specific topics, activities, or learning experiences to specific time slots within the timetable.
Delivery of content	It specifies whether the sessions are delivered as isolated subject-based sessions or the teaching sessions are planned to provide a multidisciplinary perspective by co-facilitation from different disciplines.
Teaching and learning strategies	These refer to the various methods and approaches used by educators to facilitate effective learning experiences for students. These strategies include interactive lectures, PBL, CBL, flipped classroom, etc.
Assessment of curricular content	The method of evaluating and measuring the knowledge, skills, and understanding acquired by students in relation to the specific content covered within a curriculum.
Boundaries of integration	The limits or parameters within which different subjects or disciplines are combined or interconnected. This includes discipline-based, horizontal integration, vertical integration, spiral integration and interprofessional education
Faculty development	The professional growth and enhancement of faculty members in their roles as educators, researchers, and leaders.

**Table-IIS:** Rubric defining five Levels of Integration.

Integration level	Representation of Harden’s integration level	Description
1. Fragmented (No Integration)	Level 1-3	Various disciplines/departments organize their teaching and assessments without considering other departments or subjects
2. Sequenced (Nominal Integration)	Level 4-5	To enhance the teaching of one subject, materials from other subjects are utilized. Within a subject, related topics are taught independently but are sequenced, ordered, and planned concurrently with other courses.
3. Correlated (Moderate Integration)	Level 6-7	The introduction of integrated teaching sessions occurs within the subject-based framework. The two disciplines agree on the planning and execution of a teaching program employing overlapping concepts or ideas as unifying components.
4. Webbed (Advanced Integration)	Level 8-9	This combines several subject areas into a single course where themes, problems, or challenges are the teaching focus. The disciplines/subjects continue to maintain their individuality and show how they enhance the knowledge and understanding of the problem or theme.
5. Interdisciplinary (Comprehensive Integration)	Level 10-11	Discipline and subject lines become less distinct. There is no reference to individual disciplines in the timetable. The content of all or the majority of the subjects would be integrated into a new course with a new menu, reflecting a higher level of integration

### Content Validation:

### Participants, materials, and procedure:

Content validity was established using the Content Validity Index (CVI) method.^16^ A convenience sample of 23 medical education experts (Master’s degree or higher, ≥3 years’ experience) participated in the content validation. Those not actively involved in curriculum design, evaluation, or renewal were excluded. Experts first rated the relevance of each domain to curriculum integration using a 4-point scale^16^,^17^ (1 = not relevant to 4 = extremely relevant). They then evaluated the ordinal scale items for each domain on three criteria: relevance to the domain (1 = not relevant to 4 = extremely relevant), representativeness of the integration level (1 = not representative to 4 = representative), and clarity (1 = not clear to 4 = clear).^17^ Open-ended comments were invited, particularly for extreme ratings.

### Data analysis:

Item-level CVI (I-CVI) was calculated for domain relevance to curricular integration, item relevance to domain, and representativeness of integration levels by dichotomizing ratings (3-4 = 1; 1-2 = 0). Items with I-CVI ≥ 0.78 were retained, those between 0.70-0.77 were revised, and items < 0.70 were removed.^17^ Scale-level CVI (S-CVI) was computed as the average I-CVI across item relevance to domains ratings. Representativeness ratings were analyzed independently to identify scale items requiring revision and were not included in the calculation of S-CVI. Content clarity was assessed using the Content Clarity Average (CCA), with values ≥3.2 indicating adequate clarity.^17^ Following quantitative analysis, the identified domains and ordinal scale anchors requiring revisions were refined based on qualitative expert feedback, in consensus with the co-authors.

### Response Process Validity:

### Participants, materials, and procedure:

The second version of the tool was refined by cognitive interviews with six faculty members utilizing verbal probing and think-aloud techniques.^14^ Medical or dental faculty actively involved in curriculum design or delivery, with at least one year of teaching experience, participated in the interviews. A pilot interview was conducted to refine probes and prompts. Interviews were audio-recorded with consent, lasted approximately 10 minutes, and were observed by a second research team member to minimize interviewer bias.

### Data analysis:

The audiotaped interviews were transcribed and segmented for further analysis. Memos were created based on notes and digital recordings. Two independent reviewers coded items as having no, minor, or major problems, following predefined categories.^14^ The statements that required further clarity were modified in consensus with co-authors.

### Phase 2- Internal Structure Validity Evidence:

### Participants, materials, and procedure:

For pilot testing of the third version of the tool, the target population consisted of medical or dental college faculty members (clinical/basic/para-clinical) involved in either curriculum design, delivery, or evaluation. Faculty members with less than three months of institutional experience were excluded. Based on recommended item-to-participant ratios for factor analysis (1:5 to 1:20), a target sample size of 140 participants was calculated for the seven-domain instrument.^14^,^17^

To collect the data, both Google Forms and paper printed forms were used. The survey comprised two sections: demographic information and the tool (TIP), where participants rated the extent of integration practices within their institution. A preliminary test survey with 10 participants identified no procedural issues. The final survey was distributed via email and WhatsApp groups. Participation was voluntary, and confidentiality was assured. Reminders were sent at one and two weeks to enhance response rates.

### Data analysis:

Descriptive statistics were computed for demographic variables using IBM SPSS Statistics (version 26, IBM Corp., USA). Internal consistency was assessed using Cronbach’s alpha, interpreted as acceptable (0.50-0.70), good (0.70-0.90), or excellent (>0.90).^17^ Although the instrument employed a 5-point ordinal scale, the data were treated as continuous for factor analysis, consistent with recommendations for variables with five or more response categories.^18^ Exploratory factor analysis (EFA) was conducted using Principal Axis Factoring with varimax rotation to examine the underlying structure of the instrument. Sampling adequacy was evaluated using the Kaiser-Meyer-Olkin (KMO) measure and Bartlett’s test of sphericity. Factors with eigenvalues >1 and item loadings ≥0.40 were retained.

Confirmatory factor analysis (CFA) was performed using IBM SPSS AMOS (version 24) to verify the factor structure. Model fit was evaluated using absolute (GFI, RMSEA), incremental (CFI, TLI, AGFI, NFI), and parsimonious (χ²/df) fit indices, with values interpreted according to established thresholds.^17^

## RESULTS

### Phase I- Development of the instrument:

### Identification of Domain and Construction of an Ordinal Scale and a Rubric:

The first version of the tool had eight domains representing constructs of curriculum integration (Supplementary file 2: Annexure-A). These included;

- curricular aims and objectives,
- consultation in course planning prior to delivery,
- representation of content in timetables,
- delivery of content,
- teaching and learning strategies,
- assessment of curricular content,
- boundaries of integration, and
- faculty development (Supplementary Table-IS).

The accompanying rubric described five levels of integration, starting from no integration to comprehensive integration (fragmented, sequenced, correlated, webbed, interdisciplinary) (Supplementary Table-IIS). Each domain was rated on a five-point ordinal scale with descriptive anchors indicating progressively higher levels of integration.

### Content Validation:

The response rate for expert validation was 74% (17/23). Domains 1-6 demonstrated acceptable content validity (I-CVI ≥ 0.78). Domain 7 showed marginal content validity (I-CVI = 0.76) and was revised and retained based on expert feedback. Domain eight (faculty development) did not meet the acceptable threshold (I-CVI = 0.64) and was therefore removed along with its associated rating scale (Supplementary file 3: Annexure 1).

For relevance to domains, all ordinal scale items demonstrated acceptable I-CVI values, except for three items that showed marginal scores (I-CVI = 0.70-0.76); these were revised and retained following expert feedback (Supplementary file 3: Annexure 2). Regarding representativeness of integration levels, most items achieved strong agreement (I-CVI > 0.83). A small number of items with borderline values (I-CVI = 0.70-0.78) were modified and retained (Supplementary file 3: Annexure 3).

All scale items demonstrated acceptable clarity (CCA ≥ 3.2), except one item in Domain 6 (CCA = 2.94), which was revised and retained through consensus (Supplementary file 3: Annexure 4). The overall scale demonstrated strong content validity (S-CVI/Avg = 0.89) and clarity (S-CCA = 3.46). Based on qualitative feedback, all retained items were reworded for clarity and consistency. The resulting second version of the tool comprised seven domains, each rated on a five-point ordinal scale (Supplementary file 2: Annexure B).

### Response Process Validity:

The revised tool, comprising seven domains and 35 ordinal rating items, was evaluated for response process validity. Thirty-one items required no modification, while four items demonstrated minor clarity issues and were revised accordingly (Supplementary file 2: Annexure C).

### Phase 2 - Internal Structure Validity Evidence:

A total of 162 faculty members from 13 medical and dental colleges participated in the pilot testing, of whom 150 provided complete responses. They represented a range of academic positions, with most being assistant professors (31%), demonstrators/lecturers (28%), or senior registrars (19%). Over half (54%) had ≤5 years of teaching experience, while nearly two-thirds (62.7%) reported involvement in curriculum development, and 46% had served as module coordinators or directors. Most had attended workshops or completed certification or master’s degree, although 53% reported no formal qualification in medical education (Supplementary file 1: Table-IIIS).

**Table-IIIS:** Demographic data of participants for factor analysis.

	Frequency (n)	Percent %
***Department***		
Basic sciences	42	28 %
Clinical sciences	99	66 %
Educational or social sciences	9	6 %
	150	100 %
***Academic Position (Designation)***		
Professor	8	5.3%
Associate Professor	23	15.3%
Assistant Professor	46	30.7%
Senior Registrar	29	19.3%
Registrar	2	1.3%
Demonstrator/Lecturer/Postgraduate Resident	42	28.0%
Total	150	100.0%
***Teaching Experience***		
More than 15 years	19	12.7%
11–15 years	14	9.3%
6–10 years	36	24.0%
Up to 5 years	81	54.0%
Total	150	100.0%
***Involvement in Curriculum Development/Renewal***		
No	33	22.0%
Sometimes	23	15.3%
Yes	94	62.7%
Total	150	100.0%
***Role as a Module Coordinator or Director***		
No	81	54.0%
Yes	69	46.0%
Total	150	100.0%
***Qualification in Medical Education***		
Certificate	47	31.3%
Diploma	11	7.3%
Masters (MHPE/MME)	37	24.7%
PhD/Doctorate	2	1.3%
None	53	35.3%
Total	150	100.0%

The dataset was suitable for factor analysis (KMO = 0.88; Bartlett’s Test of Sphericity: χ²(150) = 473.40, p < 0.001). Exploratory factor analysis using principal axis factoring identified a single-factor solution with eigenvalues >1, accounting for 51.56% of the total variance. All domains demonstrated satisfactory factor loadings (0.61-0.79), and the scree plot supported the one-factor structure (Supplementary file 1: Fig.1s; Table-IVS & VS). Internal consistency of the tool was high (Cronbach’s alpha = 0.88). Corrected item-to-total correlations exceeded 0.40 for all domains, indicating good internal reliability (Table-I).

**Table-IVS:** Factor Matrix and Rotated Factor Matrix.

Factor Matrix^a^	
	Factor
	1
Domain 4: Delivery of content	.790
Domain 2: Course Planning prior to course delivery	.778
Domain 3: Representation of content in timetables	.753
Domain 1: Curricular Aims and objectives/outcomes	.707
Domain 7: Boundaries of integration (integrated curriculum)	.685
Domain 5: Teaching and learning strategies	.683
Domain 6: Assessment of curricular content	.614
Extraction Method: Principal Axis Factoring.	
a. 1 factors extracted. 5 iterations required.	
Rotated Factor Matrix^a^	
a. Only one factor was extracted. The solution cannot be rotated.	

**Table-VS:** Total Variance explained.

Factor	Initial Eigenvalues			Extraction Sums of Squared Loadings		
	Total	% of Variance	Cumulative %	Total	% of Variance	Cumulative %
1	4.083	58.324	58.324	3.609	51.562	51.562
2	.794	11.336	69.661			
3	.618	8.835	78.495			
4	.442	6.312	84.807			
5	.396	5.655	90.462			
6	.353	5.044	95.506			
7	.315	4.494	100.000			

***Extraction Method:*** Principal Axis Factoring.

**Table-I:** Reliability statistics of Individual Domains.

	Corrected Item-Total Correlation	Squared Multiple Correlation	Cronbach’s Alpha if Item Deleted
Domain 1: Curricular Aims and objectives/outcomes	.658	.469	.864
Domain 2: Course Planning prior to course delivery	.723	.566	.855
Domain 3: Representation of content in timetables	.703	.543	.858
Domain 4: Delivery of content	.733	.546	.854
Domain 5: Teaching and learning strategies	.637	.469	.866
Domain 6: Assessment of curricular content	.570	.370	.874
Domain 7: Boundaries of integration (integrated curriculum)	.636	.467	.866

Confirmatory factor analysis further supported the unidimensional structure of TIP. The measurement model demonstrated strong standardized regression weights (0.60-0.80). All standardized residual covariances were below 2, indicating no significant additional covariance between the observed variables (Supplementary file 1: Table-VIS). Overall, CFA fit indices reflected an excellent good model fit (Fig.2), confirming the factor structure identified through EFA (Supplementary file 1: Table-VIIS). The final version of the tool (TIP) is presented in Table-II.

**Table-VIS:** Standardized Residual Covariance values.

	Domain7	Domain6	Domain5	Domain4	Domain3	Domain2	Domain1
Domain7	.000						
Domain6	.898	.000					
Domain5	.000	1.138	.000				
Domain4	.250	.697	.083	.000			
Domain3	-.497	-.097	-.536	-.213	.000		
Domain2	-.015	-.699	-.144	-.231	.482	.000	
Domain1	-.195	-.790	.135	.059	.131	.161	.000

**Table-VIIS:** Summary of Results for Model Fit.

Measure	Estimate	Threshold*	Interpretation
χ 2	18.237	--	--
DF	13	--	--
P-value	0.149	>0.05	Excellent
χ 2 /df	1.403	Between 1 and 3	Excellent
CFI	0.989	>0.95	Excellent
TLI	0.98	>0.95	Excellent
GFI	0.966	>0.95	Excellent
AGFI	0.92	>0.90	Excellent
NFI	0.96	>0.95	Excellent
RMSEA	0.052	<0.05-0.08	Acceptable
P Close	0.429	>0.05	Excellent

**Table-II:** Final Version of the Tool of Integration Practices (TIP) with Rating Indicators and Rubric.

*Domain 1: Curricular Aims and objectives/outcomes*	
1	Learning objectives are specific to each discipline
2	Each discipline develops learning objectives in consultation with other subjects so that other subjects are aware of them.
3	Individual disciplines recognize the broader curriculum outcomes and relate their teaching plan to these, like; communication and problem-solving skills, professionalism.
4	Multiple disciplines come together to offer a joint course with themes that emphasize overall curriculum outcomes (knowledge, skills, and attitude).
5	Curriculum outcomes represent the application of knowledge to real-world problems with no reference to individual disciplines.
***Domain 2: Course Planning prior to course delivery***	
1	Course planning occurs in isolation and without consideration of other disciplines
2	Course planning occurs in isolation, but Instructors are made aware of what is covered in other disciplines to avoid unnecessary duplication
3	Consultation in course planning occurs between a few disciplines
4	Course Planning and designing occur through collaboration between experts in multiple disciplines, but subject boundaries are preserved
5	Courses are planned around integrating themes or problems that dissolve subject boundaries
***Domain 3: Representation of content in timetables***	
1	Individual sessions are labelled with the names of the subjects across the timetable
2	Similar topics are aligned together within the timetable; however, each session is labelled with an individual subject
3	Topics are aligned under themes in the timetable, with contributions of individual subjects as separate sessions
4	Topics are aligned under themes in the timetable, with major contributions of individual subjects in integrated sessions
5	Topics are aligned under themes in the timetable, having an integrated session with no identification of individual subjects
***Domain 4: Delivery of content***	
1	The session is delivered in isolation without consideration of other disciplines
2	Separate instructional sessions are delivered for related concepts across disciplines, but with coordinated timing of delivery (same day or week)
3	Separate coordinated instructional sessions are delivered, along with some sessions in co-facilitation with related disciplines
4	The majority of teaching sessions are in co-facilitation to provide a multidisciplinary application of content, along with some discipline-based sessions
5	All the content is delivered through co-facilitation in shared instructional methods and activities
***Domain 5: Teaching and learning strategies***	
1	Concepts are taught in subject-based teaching sessions (lectures, SGD, practical)
2	Subject-based teaching sessions (lectures, SGD, practical) that incorporate related concepts of the other subjects
3	Integrated teaching sessions (e.g, PBL, CBL, TBL) are introduced in addition to subject-based teaching
4	Integrated teaching sessions (e.g, PBL, CBL, TBL) are a major feature (almost 50%) of the curriculum as a teaching strategy
5	Integrated teaching sessions (almost 100%) with loss of disciplines’ identity
***Domain 6: Assessment of curricular content***	
1	Subject-based assessment of knowledge (using MCQ /SAQs/SEQs) and practical/clinical skills
2	Subject-based assessment of knowledge (using MCQ /SAQs/SEQs) and practical/clinical skills, along with limited competency assessments
3	Mainly subject and competency-based exams of knowledge, skill, and attitude, along with assessment of integrated content (through Case cluster MCQs, EMQs, integrated OSCE, portfolios, etc
4	Equal weightage of subject-based competency assessments and assessment of integrated content (through Case cluster MCQs, EMQs, integrated OSCE, portfolios, etc)
5	Completely Integrated assessments
***Domain 7: Boundaries of integration (integrated curriculum)***	
1	Discipline based
2	Discipline-based with limited horizontal integration (integrated modules within a semester or year)
3	Mainly horizontal integration, along with some vertical integration (longitudinal integrated modules in multiple years)
4	Complete horizontal and vertical integration with or without Spiral integration (integration across years and subjects)
5	Spiral integration, along with Interprofessional exposure (involving 2 or more professions)

***Integration score:*** All seven domains are marked on their respective scale, given above (with values ranging from 1 to 5), indicating the integration practices for each domain. The Final integration score is calculated as a mean value of the above scores, thus indicating the overall level of integration for the curriculum under consideration, according to the rubric given below.

**Table:** 

Rubric			
Integration score (mean)	Integration level	Representation Harden’s Integration level	Description
1-1.5	Fragmented (No Integration)	Level 1-3	Various disciplines/departments organize their teaching and assessments without considering other departments or subjects
>1.5- 2.5	Sequenced (Nominal Integration)	Level 4-5	To enhance the teaching of one subject, materials from other subjects are utilized. Within a subject, related topics are taught independently but are sequenced, ordered, and planned concurrently with other courses.
>2.5-3.5	Correlated (Moderate Integration)	Level 6-7	The introduction of integrated teaching sessions occurs within the subject-based framework. The two disciplines agree on the planning and execution of a teaching program employing overlapping concepts or ideas as unifying components.
>3.5-4.5	Webbed (Advanced Integration)	Level 8-9	This combines several subject areas into a single course where themes, problems, or challenges are the teaching focus. The disciplines/subjects continue to maintain their individuality and show how they enhance the knowledge and understanding of the problem or theme.
>4.5-5	Interdisciplinary (Comprehensive Integration)	Level 10-11	Discipline and subject lines become less distinct. There is no reference to individual disciplines in the timetable. The content of all or the majority of the subjects would be integrated into a new course with a new menu, reflecting a higher level of integration.

**Fig.2:**
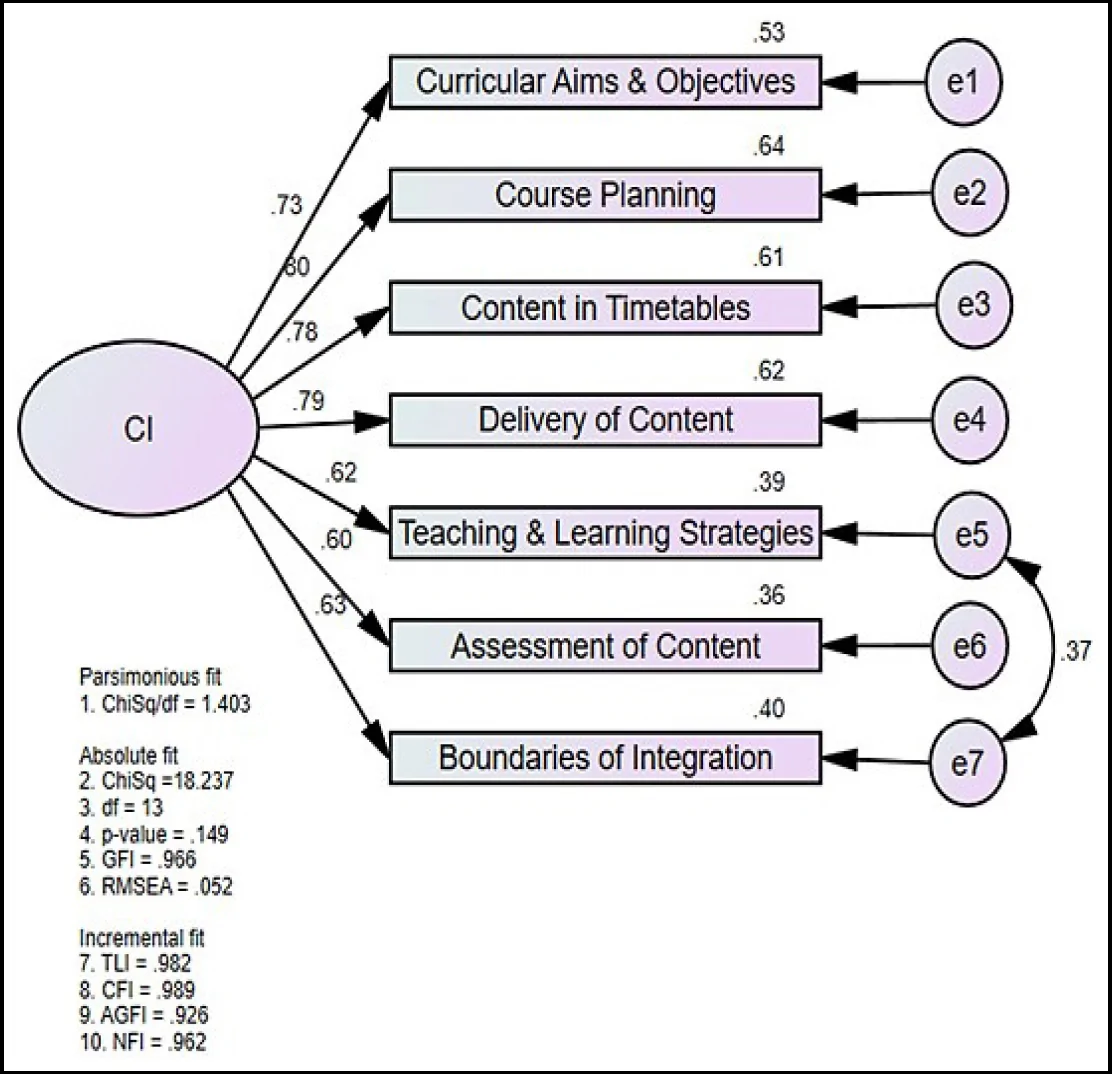
Sequential Equation Model for Instrument.

**Fig.1S:**
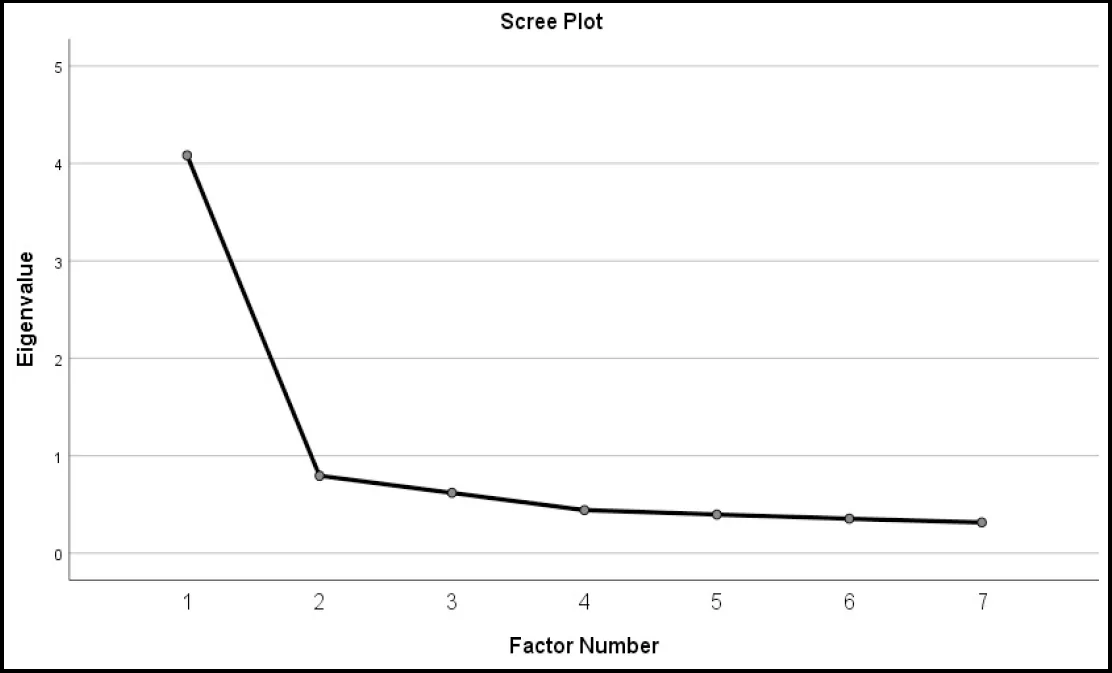
Scree plot.

The figure shows factor loadings, factor correlations, and good fit indices (parsimonious, absolute, and incremental fit) for a one-factor model containing 7 items. Abbreviations used: CI = curriculum integration, AGFI = adjusted goodness of fit index, CFI = comparative fit index, GFI = goodness-of-fit index, NFI = normed fit index, RMSEA = root mean square error of approximation, TLI = tucker lewis index, χ 2 /df = chi-square difference.

## DISCUSSION

In this study, we developed and validated the Tool of Integration Practices (TIP), a seven-domain instrument designed to assess the level of integration within health professions curricula. The instrument demonstrated good internal consistency (Cronbach’s α = 0.88) along with evidence of content, response process, and internal structure validity, supporting its use as a reliable framework for evaluating integration at both course and program levels.

The seven domains identified in TIP reflect key stages at which integration is conceptualized, implemented, and evaluated within curricula.^2^ These domains were derived from established theories of curriculum integration and align with prior conceptual models emphasizing integration as a continuum rather than a dichotomous state.^2^,^12^,^13^ Unlike previous frameworks, TIP recognizes that integration may vary across curricular domains, enabling domain-specific assessment rather than assuming uniform progression across the curriculum.^3^,^4^,^12^ The five integration levels were deliberately structured as a simple ordinal continuum with clearly differentiated descriptors, making them easier to understand, interpret, recall, and apply consistently by educators and curriculum leaders.

TIP retains the conceptual strengths of Harden’s framework while addressing its limited applicability for systematic evaluation.^3^,^4^ By assessing faculty perceptions of observable integration practices, TIP captures both disciplinary relationships and the curricular processes that support and sustain integration, offering a practical and psychometrically informed approach for curriculum assessment. This is particularly relevant for institutions that are undergoing curricular transitions.^1^,^7^,^10^,^19^,^20^ Moreover, this instrument does not assume that curricula progress through a fixed hierarchy of stages, acknowledging that individual domains may reach various levels of integration at any given time, depending on institutional priorities and context.^3^

Compared to the existing tools, TIP offers distinct advantages.^21^,^22^ Previous tools have primarily relied on faculty perceptions using generic Likert-type response scales or have been limited to evaluating individual teaching sessions.^21^,^23^ In contrast, TIP incorporates domain-specific descriptive rating anchors, which reduce ambiguity, improve response consistency, and facilitate more meaningful interpretation of integration practices.^23^ Moreover, in comparison to the Session Integration Tool (SIT) by Heck and Chase,^22^ it extends beyond session-level evaluation to the program level, offering a standardized, psychometrically tested framework for curricular assessment.

### Strength:

A major strength of this study is the rigorous development and validation of TIP using Messick’s unified framework for validity.^14^ To our knowledge, TIP is the first psychometrically validated instrument that utilizes domain-specific descriptive rating anchors and a structured scoring rubric to record faculty perceptions for comprehensive evaluation of curriculum integration at the course and program. Furthermore, the inclusion of an adequate number of experts for content validation and a robust participant-to-item ratio for factor analysis enhances the study’s credibility and methodological rigor.

The development of this novel tool, “TIP,” holds significant implications for health professions education as curriculum committees, accreditation bodies, and educational leaders can use this framework to monitor curricular implementation, identify strengths and gaps in integration practices, and conduct evidence-based curricular reviews to enhance transparency.

### Limitations:

Tool validation was conducted within institutions from a single country, which may limit generalizability to other educational settings. Moreover, this is a self-assessment tool and is subject to respondent bias. Expert validation was conducted using a single-round process rather than a Delphi technique. Additionally, variability in rubric interpretation may occur across evaluators, requiring calibration sessions in future applications.

## CONCLUSION

The Tool of Integration Practices (TIP) is a valid and reliable instrument for assessing integration practices based on faculty perceptions within medical and dental curricula. It demonstrated strong evidence of content, response process, and internal structure validity. TIP provides a practical framework for evaluating integration at the course and program levels and may support evidence-informed curriculum evaluation and development.

### Recommendations:

Future investigation should involve the practical application of this tool across diverse institutional and cultural contexts to establish its generalizability and utility.
